# Supplementary material for: Epidemiology and care management of Paroxysmal Nocturnal Hemoglobinuria (PNH) in a real-world setting in France: Description from the French National Hospitalization Database
Source: PLoS One. 2026 Apr 16;21(4):e0339715. doi: 10.1371/journal.pone.0339715 (PMC13086315; doi:10.1371/journal.pone.0339715)
Supplement: S1 File — (DOCX) [file pone.0339715.s001.docx]

**S1 Fig. Study design**


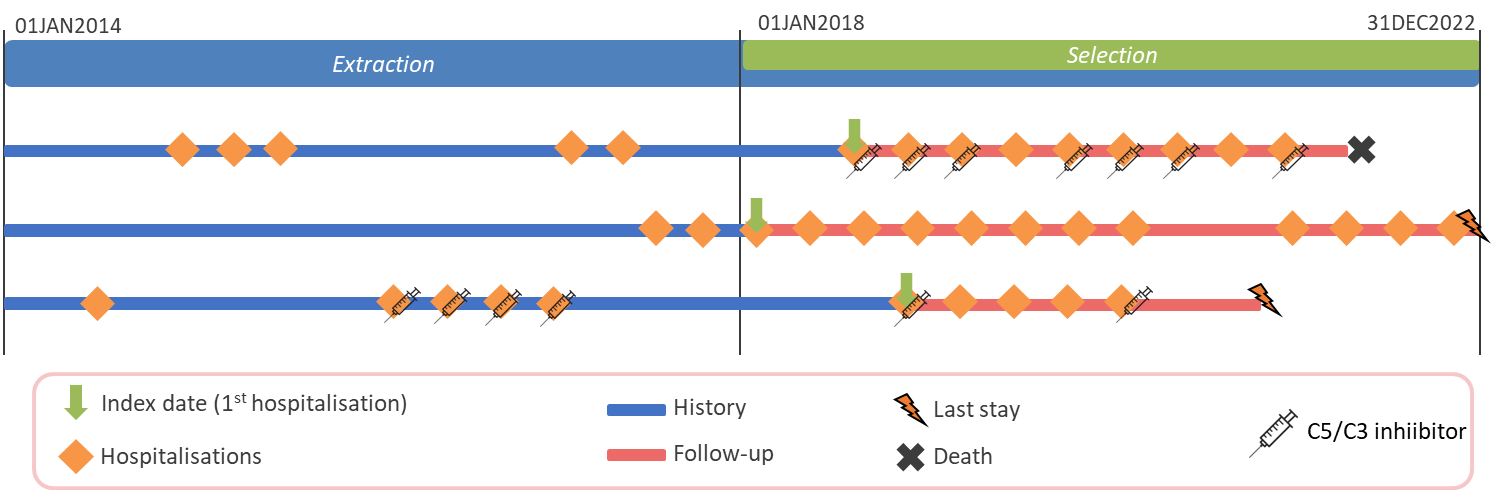


**S1 Table. Indications available for reimbursement as « liste en sus » therapy for C5 inhibitors**

| Drug | Indication | Inclusion |
| --- | --- | --- |
| Eculizumab | Treatment of neuromyelitis optica spectrum disorder in adult patients who are positive for anti–aquaporin-4 antibodies, with the recurrent form of the disease (2 attacks in the past year or 3 attacks in the past two years, including at least one in the previous year), who have had an inadequate response to background immunosuppressive therapies (rituximab, azathioprine, mycophenolate mofetil). | NO |
|  | In adults and children for the treatment of patients with atypical Hemolytic Uremic Syndrome (aHUS). | NO |
|  | In adults and children with a history of transfusion, for the treatment of patients with Paroxysmal Nocturnal Hemoglobinuria (PNH). Clinical benefit has been demonstrated in patients who exhibit hemolysis with one or more clinical symptom(s) indicating high disease activity, regardless of transfusion history. | YES |
| Ravulizumab | Treatment of paroxysmal nocturnal hemoglobinuria (PNH) in pediatric patients weighing 10 kg or more:  - who have hemolysis with one or more clinical symptom(s) indicating high disease activity;  - who are clinically stable following treatment with eculizumab for at least the past 6 months. | YES |
|  | Treatment of paroxysmal nocturnal hemoglobinuria (PNH) in adult patients:  - who have hemolysis with one or more clinical symptom(s) indicating high disease activity;  - who are clinically stable following treatment with eculizumab for at least the past 6 months. | YES |

(see <https://sante.gouv.fr/soins-et-maladies/medicaments/professionnels-de-sante/autorisation-de-mise-sur-le-marche/la-liste-en-sus/article/referentiel-des-indications-des-specialites-pharmaceutiques-inscrites-sur-la>)

**S2 Table. Selection criteria**

| Inclusion criteria | Patients with at least one hospitalisation (PMSI MCO-HAD) with a diagnosis code, as main, related or associated diagnosis, based on ICD-10 during the selection period (2018-2022): D59.5 Paroxysmal nocturnal haemoglobinuria [Marchiafava-Micheli]  AND/OR  Patients with at least one dispensation of anti-C5 antibody, based on ATC codes:   \| L04AA25 \| Eculizumab \| \| --- \| --- \| \| L04AA43 \| Ravulizumab \| |
| --- | --- | --- | --- | --- | --- |
| Exclusion criteria | Patients with hospitalisation (PMSI MCO-HAD) with main, related ~~or associated diagnoses~~, based on ICD-10 during the selection period (2018-2022):   \| D59.3 \| Haemolytic-uraemic syndrome \| \| --- \| --- \| \| G70 \| Myasthenia gravis and other myoneural disorders \| \| G36.0 \| Neuromyelitis optica \| \| G36.8 \| Other specified demyelinating diseases of central nervous system \| \| G36.9 \| Demyelinating disease of central nervous system, unspecified \|   Initial algorithm will consider **AT LEAST ONE** hospitalisation with a code described above. Further algorithms will consider **AT LEAST X** hospitalisations, with a progressive increase.  Final algorithm will be selected based on the number and characteristics of patients after discussions with Roche and the independent scientific reviewer. |
| Subgroups | Among patients from the study population, the following subgroups will be described:  Patients treated with anti-C5 antibodies, corresponding to patients presenting at least one dispensation of anti-C5 antibody (PMSI MCO-HAD), based on ATC codes during the selection period (2018-2022):   \| L04AA25 \| Eculizumab \| \| --- \| --- \| \| L04AA43 \| Ravulizumab \|   Patients initiating a treatment with anti-C5, corresponding to patients presenting at least one dispensation of anti-C5 antibody (see above) during the selection period (2018-2022)  AND with no previous dispensation of anti-C5 antibody during the historical period (up to 2013)  Patients with a switch of anti-C5, corresponding to patients treated with anti-C5 (see above), presenting at least one change of ATC code between eculizumab and ravulizumab |
| C3 inhibitor | ATC code   \| L04AA54 \| Pegcetacoplan \| \| --- \| --- \| |

**S3 Table. Medical history**

| Renal failure | Patients presenting at least one of the following conditions over the historical period  Patients with stays including at least one of the following **ICD-10 codes,** as main, related or associated diagnosis (PMSI MCO-HAD)**:**   \| N18 \| Chronic kidney disease \| \| --- \| --- \| \| Y84.1 \| Kidney dialysis \| \| Z99.2 \| Dependence on renal dialysis \|   AND/OR  At least one medical procedure for which the **CCAM code** belongs to the following list (PMSI MCO):   \| JVJB001 \| *Séance d'épuration extrarénale par dialyse péritonéale pour insuffisance rénale chronique* \| \| --- \| --- \| \| JVJF004 \| *Séance d'épuration extrarénale par hémodialyse pour insuffisance rénale chronique* \| \| JVJF008 \| *Séance d'épuration extrarénale par hémodiafiltration, hémofiltration ou biofiltration sans acétate pour insuffisance rénale chronique* \| \| JVRP004 \| *Séance d'entraînement à l'hémodialyse* \| \| JVRP007 \| *Séance d'entraînement à la dialyse péritonéale automatisée* \| \| JVRP008 \| *Séance d'entraînement à la dialyse péritonéale continue ambulatoire* \| \| YYYY007 \| *Forfait hebdomadaire de prise en charge d'un patient insuffisant rénal en dialyse péritonéale* \|   AND/OR  At least one of the following **GHM codes**:   \| 11K021 \| *Insuffisance rénale, avec dialyse, niveau 1* \| \| --- \| --- \| \| 11K022 \| *Insuffisance rénale, avec dialyse, niveau 2* \| \| 11K023 \| *Insuffisance rénale, avec dialyse, niveau 3* \| \| 11K024 \| *Insuffisance rénale, avec dialyse, niveau 4* \| \| 11K02J \| *Insuffisance rénale, avec dialyse, en ambulatoire* \| \| 28Z01Z \| *Entraînements à la dialyse péritonéale automatisée, en séances* \| \| 28Z02Z \| *Entraînements à la dialyse péritonéale continue ambulatoire, en séances* \| \| 28Z03Z \| *Entraînements à l'hémodialyse, en séances* \| \| 28Z04Z \| *Hémodialyse, en séances* \| \| 28Z05Z \| *Dialyse péritonéale automatisée, en séances* \| \| 28Z06Z \| *Dialyse péritonéale continue ambulatoire, en séances* \|   AND/OR  At least one **fee** (i.e. variable GHS_NUM) (PMSI-MCO) **or external procedure** of the following list:   \| D01 \| *Hémodialyse en centre ou en unité de dialyse médicalisée* \| \| --- \| --- \| \| D02 \| *Autodialyse simple ou assistée* \| \| D03 \| *Entraînement à l’hémodialyse à domicile et à l’autodialyse* \| \| D04 \| *Entraînement à la DPA* \| \| D05 \| *Entraînement à la DPCA* \| \| D06 \| *Hémodialyse à domicile* \| \| D07 \| *Dialyse péritonéale automatisée (DPA)* \| \| D08 \| *Dialyse péritonéale continue ambulatoire (DPCA)* \| \| D09 \| *Forfait d’hémodialyse en centre* \| \| D10 \| *Forfait d’hémodialyse en centre pour enfant* \| \| D11 \| *Forfait d’hémodialyse en unité de dialyse médicalisée* \| \| D12 \| *Forfait d’autodialyse simple* \| \| D13 \| *Forfait d’autodialyse assistée* \| \| D14 \| *Forfait d’autodialyse à domicile* \| \| D15 \| *Forfait de dialyse péritonéale automatisée (DPA)* \| \| D16 \| *Forfait de dialyse péritonéale continue ambulatoire (DPCA)* \| \| D17 \| *Forfait d’entraînement à l’hémodialyse à domicile et autodialyse* \| \| D18 \| *Forfait d’entraînement à la dialyse péritonéale automatisée* \| \| D19 \| *Forfait d’entraînement à la dialyse péritonéale continue ambulatoire* \| \| D20 \| *Forfait d'entraînement à la dialyse péritonéale automatisée à domicile* \| \| D21 \| *Forfait d'entraînement à dialyse péritonéale continue ambulatoire à domicile* \| \| D22 \| *Forfait de dialyse péritonéale automatisée pour hospitalisation de 3 à 6 jours* \| \| D23 \| *Forfait de dialyse péritonéale continue ambulatoire pour hospitalisation de 3 à 6 jours* \| \| D24 \| *Forfait d’entraînement à l’hémodialyse en unité de dialyse médicalisé* \| \| DIP \| *Supplément dialyse péritonéale* \|   AND/OR  At least one **hospitalisation** (PMSI-MCO) with:   - **SUP_ENT_DPA =1** - **And/or SUP_ENT_DPC = 1** - **And/or SUP_ENT_HEM = 1**   **And/or SUP_HEM_HS = 1** |
| --- | --- | --- | --- | --- | --- | --- | --- | --- | --- | --- | --- | --- | --- | --- | --- | --- | --- | --- | --- | --- | --- | --- | --- | --- | --- | --- | --- | --- | --- | --- | --- | --- | --- | --- | --- | --- | --- | --- | --- | --- | --- | --- | --- | --- | --- | --- | --- | --- | --- | --- | --- | --- | --- | --- | --- | --- | --- | --- | --- | --- | --- | --- | --- | --- | --- | --- | --- | --- | --- | --- | --- | --- | --- | --- | --- | --- | --- | --- | --- | --- | --- | --- | --- | --- | --- | --- | --- | --- | --- | --- | --- | --- | --- |
| Hepatic failure | Patients with stays including at least one of the following **ICD-10 codes** as main, related or associated diagnosis over historical period   \| B18 \| Chronic viral hepatitis \| \| --- \| --- \| \| I85 \| Oesophageal varices \| \| K70 \| Alcoholic liver disease \| \| K71 \| Toxic liver disease \| \| K72 \| Hepatic failure, not elsewhere classified \| \| K73 \| Chronic hepatitis, not elsewhere classified \| \| K74 \| Fibrosis and cirrhosis of liver \| \| K75 \| Other inflammatory liver diseases \| \| K76 \| Other diseases of liver \| \| Z94.4 \| Liver transplant status \| |
| Stroke | Patients with stays including at least one of the following **ICD-10 codes** as main, related or associated diagnosis over historical period   \| I60 \| Subarachnoid haemorrhage \| \| --- \| --- \| \| I61 \| Intracerebral haemorrhage \| \| I62 \| Other nontraumatic intracranial haemorrhage \| \| I63 \| Cerebral infarction \| \| I64 \| Stroke, not specified as haemorrhage or infarction \| \| I67 \| Other cerebrovascular diseases \| \| I68 \| Cerebrovascular disorders in diseases classified elsewhere \| \| I69 \| Sequelae of cerebrovascular disease \| |
| Myocardial infarction | Patients with stays including at least one of the following **ICD-10 codes** as main, related or associated diagnosis over historical period   \| I20 \| Angina pectoris \| \| --- \| --- \| \| I21 \| Acute myocardial infarction \| \| I22 \| Subsequent myocardial infarction \| \| I23 \| Certain current complications following acute myocardial infarction \| \| I24 \| Other acute ischaemic heart diseases \| \| I25 \| Chronic ischaemic heart disease \| |
| Pulmonary embolism | Patients with stays including at least one of the following **ICD-10 codes** as main, related or associated diagnosis over historical period   \| I26 \| Pulmonary embolism \| \| --- \| --- \| |
| Other neuro-cardiovascular thrombotic disease | Patients with stays including at least one of the following **ICD-10 codes** as main, related or associated diagnosis over historical period   \| I05 \| Rheumatic mitral valve diseases \| \| --- \| --- \| \| I06 \| Rheumatic aortic valve diseases \| \| I07 \| Rheumatic tricuspid valve diseases \| \| I08 \| Multiple valve diseases \| \| I09 \| Other rheumatic heart diseases \| \| I11 \| Hypertensive heart disease \| \| I12 \| Hypertensive renal disease \| \| I13 \| Hypertensive heart and renal \| \| I27 \| Other pulmonary heart diseases \| \| I30 \| Acute pericarditis \| \| I31 \| Other diseases of pericardium \| \| I32 \| Pericarditis in diseases classified elsewhere \| \| I33 \| Acute and subacute endocarditis \| \| I34 \| Nonrheumatic mitral valve disorders \| \| I35 \| Nonrheumatic aortic valve disorders \| \| I36 \| Nonrheumatic tricuspid valve disorders \| \| I37 \| Pulmonary valve disorders \| \| I38 \| Endocarditis, valve unspecified \| \| I39 \| Endocarditis and heart valve disorders in diseases classified elsewhere \| \| I40 \| Acute myocarditis \| \| I41 \| Myocarditis in diseases classified elsewhere \| \| I42 \| Cardiomyopathy \| \| I43 \| Cardiomyopathy in diseases classified elsewhere \| \| I44 \| Atrioventricular and left bundle-branch block \| \| I45 \| Other conduction disorders \| \| I46 \| Cardiac arrest \| \| I47 \| Paroxysmal tachycardia \| \| I48 \| Atrial fibrillation and flutter \| \| I49 \| Other cardiac arrhythmias \| \| I50 \| Heart failure \| \| I51 \| Complications and ill-defined descriptions of heart disease \| \| I52 \| Other heart disorders in diseases classified elsewhere \| \| I70 \| Atherosclerosis \| \| I71 \| Aortic aneurysm and dissection \| \| I72 \| Other aneurysm and dissection \| \| I73 \| Other peripheral vascular diseases \| \| I74 \| Arterial embolism and thrombosis \| \| I77 \| Other disorders of arteries and arterioles \| \| I78 \| Diseases of capillaries \| \| I79 \| Disorders of arteries, arterioles and capillaries in diseases classified elsewhere \| \| I80 \| Phlebitis and thrombophlebitis \| \| I81 \| Portal vein thrombosis \| \| I82 \| Other venous embolism and thrombosis \| \| I89 \| Other noninfective disorders of lymphatic vessels and lymph nodes \| \| I98 \| Other disorders of circulatory system in diseases classified elsewhere \| \| I99 \| Other and unspecified disorders of circulatory system \| |
| Diabetes | Patients with stays including at least one of the following **ICD-10 codes** as main, related or associated diagnosis over historical period   \| E10 \| Type 1 diabetes mellitus \| \| --- \| --- \| \| E11 \| Type 2 diabetes mellitus \| \| E12 \| Malnutrition-related diabetes mellitus \| \| E13 \| Other specified diabetes mellitus \| \| E14 \| Unspecified diabetes mellitus \| \| G59.0 \| Diabetic mononeuropathy \| \| G63.2 \| Diabetic polyneuropathy \| \| G73.0 \| Myasthenic syndromes in endocrine diseases \| \| G99.0 \| Autonomic neuropathy in endocrine and metabolic diseases \| \| H28.0 \| Diabetic cataract \| \| H36.0 \| Diabetic retinopathy \| \| I79.2 \| Peripheral angiopathy in diseases classified elsewhere \| \| L97 \| Ulcer of lower limb, not elsewhere classified \| \| M14.2 \| Diabetic arthropathy \| \| M14.6 \| Neuropathic arthropathy \| \| N08.3 \| Glomerular disorders in diabetes mellitus \| |

**S4 Table. Reference and competence centers for PNH**

| FINESS Identifier | Center | Type |  |
| --- | --- | --- | --- |
| 75 080 345 4 | APHP Hôpital Robert Debré – Paris | Reference |  |
| 75 010 007 5 | APHP Hôpital Saint Louis – Paris | Reference |  |
| 59 081 127 9 | Hôpital Claude Huriez – CHRU Lille | Competence |  |
| 59 000 660 7 | Hôpital Jeanne de Flandre – CHRU Lille | Competence |  |
| 33 078 364 8 | Hôpital Haut Lévèque – CHU Bordeaux | Competence |  |
| 69 078 413 7 | HCL Hôpital Lyon Sud – Lyon | Competence |  |
| 13 078 329 3 | APHM Hôpital de la Timone – Marseille | Competence |  |
| 13 000 164 7 | Institut Paoli Calmettes – Marseillle | Competence |  |
| 97 021 121 5 | CHU de Martinique – Fort de France | Competence | |
| 97 040 858 9 | CHU Sud Réunion – Saint-Pierre – Réunion | Competence | |
