# Supplementary material for: Epidemiology and care management of Paroxysmal Nocturnal Hemoglobinuria (PNH) in a real-world setting in France: Description from the French National Hospitalization Database
Source: PLoS One. 2026 Apr 16;21(4):e0339715. doi: 10.1371/journal.pone.0339715 (PMC13086315; doi:10.1371/journal.pone.0339715)
Supplement: Attachment — Submitted filename: Human_Subjects_Research_Checklist_HPN_PMSI.docx [file pone.0339715.s002.docx]

**Human Participants Research Checklist**

***Complete the following if your study involved human participants or human participants’ data. These questions should be addressed for prospective and retrospective studies.***

1. Did you obtain ethics approval for this study?
   - If yes, please upload (file type “Other”) all the approval documents you received from your ethics committee to cover the entire range of the study period (i.e. the original approval document and any extension documents). Where ethics approval was obtained from more than one study location, please provide approval document(s) from all of the sites. If the original document is in another language, please also provide an English translation.

**Not applicable – see Research involving human participants, lines 317-331**

- If you did not obtain ethical approval, please explain why this was not required below.

The study has been conducted in accordance with International Society for Pharmacoepidemiology guidelines for Good Pharmacoepidemiology Practices and falls under the French Data Protection Act, as described in The French Data Protection Agency (Commission Nationale de l'Informatique et des Libertés – CNIL) opinion n°2018-257 of 7 June 2018 on regulatory requirements and governance for processing PMSI data under expedited regulatory process (MR-006 – see https://www.legifrance.gouv.fr/jorf/id/JORFTEXT000037187571).

As per MR-006 process, this study only uses anonymized secondary data from claims database, qualifying as research not involving human subjects and thus no informed consent or review by an ethics committee is required. Studies carried out using PMSI data must be audited to ensure their compliance with authorized purposes. Patients retain the right to withdraw or object by submitting an explicit request to their local insurance branch (CPAM), in accordance with article 111 of the application decree n° 2019-536 of 29 May 2019, under the Data Protection Act. Detailed instructions for this process are available at: https://www.health-data-hub.fr/politique-de-confidentialite. A collective information note has been added to both stève consultants and Roche websites for this purpose.

2. If you prospectively recruited human participants for the study – for example, you conducted a clinical trial, distributed questionnaires, or obtained tissues, data or samples for the purposes of this study, please report in the Methods:
   i. the day, month and year of the **start and end** of the recruitment period for this study.
   ii. whether participants provided informed consent, and if so, what type was obtained (for instance, written or verbal, and if verbal, how it was documented and witnessed). If your study included minors, state whether you obtained consent from parents or guardians. If the need for consent was waived by the ethics committee, please include this information.

Please state the line number(s) in the Methods where this is reported ______

**Not applicable, as per MR-006 process – see Research involving human participants, lines 317-331**

3. If you are reporting a retrospective study of, for example, medical records, archived samples, survey data, please report in the Methods section:
i. the day, month and year when the data were accessed for research purposes
ii. whether authors had access to information that could identify individual participants during or after data collection

Please state the line number(s) in the Methods where this is reported

**see Research involving human participants, lines 317-331**
