# Supplementary material for: Epidemiology and care management of Paroxysmal Nocturnal Hemoglobinuria (PNH) in a real-world setting in France: Description from the French National Hospitalization Database
Source: PLoS One. 2026 Apr 16;21(4):e0339715. doi: 10.1371/journal.pone.0339715 (PMC13086315; doi:10.1371/journal.pone.0339715)
Supplement: Attachment — Submitted filename: PlOs_Mansucript_PNH_ReviewersResponses_20260219.docx [file pone.0339715.s003.docx]

**ID**: PONE-D-25-54111

**Title**: Epidemiology and care management of Paroxysmal Nocturnal Hemoglobinuria (PNH) in a Real-World Setting in France: Description from the French National Hospitalization Database

Dear Editor, dear Reviewers

Thank you for your feedback on our manuscript, " Epidemiology and care management of Paroxysmal Nocturnal Hemoglobinuria (PNH) in a Real-World Setting in France: Description from the French National Hospitalization Database ". Please find below a point-by-point response to the comments you provided.

Thank you for your consideration, and please let us know if you have any questions or require further information.

Sincerely,

Julien Vercruyssen

Medical Evidence Project Specialist

Roche France

4 Cr de l'Île Seguin

92100 Boulogne-Billancourt – France

julien.vercruyssen@roche.com

**Additional Journal Requirements**

Thank you for stating the following in the Competing Interests section:
“RPL received honoraria from Novartis, Amgen, Jazz pharmaceuticals, MSD, Sanofi, Pfizer, Swedish orphan biovitrum, Alexion, Roche
SP, JV, MDB , DP and NG are employees of Roche SAS
SB and LC are employees of stève consultants – a Cytel company, which has a research contract with Roche SAS”
Please also provide an updated Competing Interests Statement declaring this commercial affiliation along with any other relevant declarations relating to employment, consultancy, patents, products in development, or marketed products, etc.
Within your Competing Interests Statement, please confirm that this commercial affiliation does not alter your adherence to all PLOS ONE policies on sharing data and materials by including the following statement: "This does not alter our adherence to  PLOS ONE policies on sharing data and materials.” (as detailed online in our guide for authors <http://journals.plos.org/plosone/s/competing-interests)> . If this adherence statement is not accurate and  there are restrictions on sharing of data and/or materials, please state these. Please note that we cannot proceed with consideration of your article until this information has been declared.
Please include a Competing Interests Statement in your cover letter. We will change the online submission form on your behalf.

Thank you for your comment, as proposed, here is the updated competing interest statement:

Funds were allocated to stève consultants, a Cytel company, under research contract with Roche France. RPL received honoraria for participation in the development and validation of the study and its results. SP, JV, MLD, DP, NG are employees of Roche France and did not receive study-specific compensations. SB, LC are employees of stève consultants, a Cytel company, and did not receive study-specific compensation.

This does not alter authors’ adherence to PLOS ONE policies on sharing data and materials.

Aside from this study, RPL received honoraria from Novartis, Amgen, Jazz pharmaceuticals, MSD, Sanofi, Pfizer, Swedish orphan biovitrum, Alexion, Roche.

Thank you for uploading your study's underlying data set. Unfortunately, the repository you have noted in your Data Availability statement does not qualify as an acceptable data repository according to PLOS's standards. At this time, please upload the minimal data set necessary to replicate your study's findings to a stable, public repository (such as figshare or Dryad) and provide us with the relevant URLs, DOIs, or accession numbers that may be used to access these data. For a list of recommended repositories and additional information on PLOS standards for data deposition, please see <https://journals.plos.org/plosone/s/recommended-repositories>.

Thank you for your comment on data sharing capacities. As specified in MR-006 process and by the French national data protection agency (CNIL), access to PMSI data, which are the only source of data used in our study, is strictly controlled.

PMSI data can only be accessed by trained professionals, who were granted study-specific authorization, after completing the MR-006 procedure. PMSI data must be stored on a secure platform, accessible only by authorized personnel, using specific tokens and one-time login. Only aggregated results can be extracted from the secured platform. Hence, no additional data can be shared.

**Journal Requirements**

Please ensure that your manuscript meets PLOS ONE's style requirements, including those for file naming

We noted in your submission details that a portion of your manuscript may have been presented or published elsewhere. [The results of this study were presented at the EHA 2024 congress] Please clarify whether this [conference proceeding or publication] was peer-reviewed and formally published. If this work was previously peer-reviewed and published, in the cover letter please provide the reason that this work does not constitute dual publication and should be included in the current manuscript.

Only a part of the results were presented, as an abstract, at the EHA 2024 congress. The manuscript presents in more details the methods and results. While a scientific committee may have reviewed and selected the abstract, no formal peer review was done. The abstract was made available in EHA 2024 abstract book (HemaSphere Volume 8: EHA2024 Hybrid Congress). Hence, this manuscript does not constitute dual publication.

Thank you for stating the following financial disclosure: “This study was funded by Roche SAS”. Please state what role the funders took in the study. If the funders had no role, please state: "The funders had no role in study design, data collection and analysis, decision to publish, or preparation of the manuscript." If this statement is not correct you must amend it as needed. Please include this amended Role of Funder statement in your cover letter; we will change the online submission form on your behalf. Thank you for stating the following in the Competing Interests section. We note that one or more of the authors are employed by a commercial company. Please provide an amended Funding Statement declaring this commercial affiliation, as well as a statement regarding the Role of Funders in your study. If your commercial affiliation did play a role in your study, please state and explain this role within your updated Funding Statement. We note that you have provided additional information within the Acknowledgements Section that is not currently declared in your Funding Statement. Please note that funding information should not appear in the Acknowledgments section or other areas of your manuscript. We will only publish funding information present in the Funding Statement section of the online submission form.

Funding statement was deleted from the manuscript as required.

Our funding statement is the following:

The study was funded by Roche France. Funds were allocated to stève consultants, a Cytel company, under research contract with Roche France. RPL received honoraria for participation in the development and validation of the study and its results. SP, JV, MLD, DP, NG are employees of Roche France and did not receive study-specific compensations.

We note that you have indicated that there are restrictions to data sharing for this study. For studies involving human research participant data or other sensitive data, we encourage authors to share de-identified or anonymized data. However, when data cannot be publicly shared for ethical reasons, we allow authors to make their data sets available upon request.

Access to PMSI database falls within the French regulation on regulatory requirements and governance for processing PMSI data under expedited regulatory process (MR-006). Only authorized personnel can access these data, on preidentified secured platforms, after the submission of a MR-006 dossier to the French data protection agency (CNIL – see Research involving human participants). No individual data can be extracted in any form, from the secure platform, only aggregated analyses and results. This as further mentioned in the Data availability statement, with links to resource websites and contacts.

**Reviewer 1**

I want to congratulate the authors on submitting this well written manuscript. I have the following queries and suggestions:

1) The data collected is from 2018-2022. It would be a good idea to include data till the year 2024-25.

Author response and action taken: Thank you for your comment regarding data updates. We acknowledge the importance of accessing the most recent data available. However, access to PMSI data is granted on a study-by-study basis. Obtaining additional data would therefore require the submission of an entirely new study, along with the associated regulatory approval process, which would substantially delay the communication of our results. We have added a paragraph to the Discussion section to emphasize the need for updated data in future research.

2) What was the treatment response on follow up. Please include the details.

Author response and action taken: Thank you for your comment regarding the need for detailed information on treatment response. In PNH, treatment response is primarily assessed through clinical presentation and biological parameters, notably PNH clone size, which are not available in the PMSI database, which is a claims database with partial clinical data (only diagnosis and procedures). As a result, treatment response could not be robustly evaluated in this study. Moreover, the primary objective of this work was to describe the organizational impact of C5 inhibitor therapies, rather than their clinical effectiveness or biological outcomes.

3) To make the data more meaningful, it would be nice to compare the responses and outcomes of eculizumab vs ravulizumab and pegcetacoplan.

Author response and action taken: Thank you for your comment regarding the comparison of therapeutic alternatives in PNH. The primary objectives of this study were to describe PNH epidemiology and to characterize the main treatment modalities from an organizational perspective, which is the most appropriate approach given the nature of the database used. Indeed, information related to treatment response and other key clinical outcomes is limited in the PMSI database, making it difficult to define relevant and reliable clinical endpoints. Moreover, changes in PNH management predominantly rely on detailed clinical and biological parameters that are not available in PMSI. As a result, conducting direct comparisons between therapies would be subject to substantial informational bias and confounding, which would limit the robustness and validity of any comparative analyses. For these reasons, we did not undertake a comparative effectiveness study and instead focused on an organizational description of PNH treatment patterns.

4) What were the various reasons for patients not opting for C5i therapy as these are nearly 50% patients.

Author response and action taken: Thank you for your comment regarding the proportion of untreated patients with PNH. Treatment decisions in PNH rely almost exclusively on symptoms and biological data (e.g., hemolysis, thrombosis, dysphagia, pain, central anemia), which cannot be directly assessed using PMSI data. Conversely, PMSI exhaustively captures reimbursed treatments for eculizumab and ravulizumab, making the risk of non‑identification of treated patients almost null. Consequently, aside from a limited risk of miscoding (further minimized by the use of exclusion diagnoses), untreated patients identified in the database are expected to present with subclinical PNH and low‑level hemolysis. Such patients typically benefit from active surveillance rather than immediate treatment initiation. According to clinical expert opinion, an untreated patient rate of approximately 50% is consistent with current clinical practice. These considerations have been further added in the Discussion section.

5) What were the reasons for patients switching back to eculizumab from ravulizumab? Kindly elaborate

Author response and action taken: Thank you for your comment regarding the need to explain why patients may switch back to eculizumab. The lack of detailed clinical information in the PMSI database limits our ability to directly assess the reasons underlying treatment changes. Nevertheless, several plausible hypotheses can be proposed. Ravulizumab was initially indicated for patients with a stable disease receiving eculizumab, mainly to reduce injection frequency. In the event of a recent complication or a severe hemolytic crisis, clinicians may consider the need for closer clinical follow‑up, including more frequent dosing, and therefore opt for a switch back to eculizumab. This may also apply to patients with specific clinical presentations or comorbidities requiring intensified monitoring. In addition, patients experiencing adverse events under ravulizumab may be considered for a switch to eculizumab. These potential explanations have been further detailed in the Discussion section.

**Reviewer 2**

This is a valuable article addressing the prevalence of PNH in France and current treatment strategies. As a minor point, in line 71, the expression “unlike the three aforementioned drugs, which are intravenous” appears to imply that pegcetacoplan is administered intravenously; however, pegcetacoplan is administered subcutaneously. Clarification or rephrasing would improve accuracy.

Author response and action taken: Thank you for your comment regarding the administration route of pegcetacoplan. The relevant paragraph has been rephrased to clearly emphasize that pegcetacoplan was administered subcutaneously, and not intravenously, as follows: “Unlike current C5 inhibitors, which are intravenous, crovalimab will be dispensed as a monthly subcutaneous injection”

**Reviewer 3**

This manuscript presents a national, real-world analysis of PNH epidemiology and hospital-based management in France using PMSI data from 2018–2022. The use of an exhaustive national hospital database is a clear strength, and the topic is timely given the evolving complement inhibitor landscape. The manuscript is generally well written and methodologically appropriate for a descriptive claims-based study. There are some key points that may benefit from revision to strengthen interpretation. In summary, this is a valuable descriptive study providing updated national data on hospital-managed PNH in France. With changes listed, the manuscript would be suitable for publication.

1) Case identification relies solely on ICD-10 code D59.5 during hospitalization. Although difficulties in epidemiological prevalence are well acknowledged. The manuscript would benefit from clearer acknowledgement on that this is PMSI-estimated prevalence. Please also clarify whether any additional criteria (e.g. repeated coding, associated procedures or treatments) were considered to increase diagnostic specificity.

Author response and action taken: Thank you for your comment regarding the definition of the study population and the associated algorithms. Prior to establishing the final, streamlined algorithm presented in the manuscript, an iterative process was conducted. This process identified three main patient groups: (i) patients with a PNH diagnosis only, (ii) patients treated with a C5 inhibitor without a recorded PNH diagnosis, and (iii) patients with both a PNH diagnosis and C5 inhibitor treatment. For each group, patients presenting exclusion diagnoses (hemolytic uremic syndrome, myasthenia gravis, neuromyelitis optica) were further stratified according to the number of exclusion diagnoses identified. The resulting potential target populations were then compared with data from literature and with clinical expert. This comparative assessment supported the validation of the selection algorithm developed and applied in the manuscript. We have added a paragraph to the Methods section to better describe this iterative approach.

2) There is limited data on PNH clone size and classification of PNH, either in the diagnostic setting or in those patients that received therapy - comment should be made if this was not available.

Author response and action taken: Thank you for your comment highlighting the importance of clinical data. As briefly mentioned in the Discussion, clinical information is limited in the PMSI database. PNH clone size and disease classification are key components of PNH management; however, they rely on data that are not available in PMSI. Specifically, biological results are not accessible, and PNH classification is based solely on the ICD-10 code D59.5. We have added a paragraph to the Discussion section to further emphasize this limitation regarding the lack of detailed clinical data.

3) It would strengthen the manuscript if there is any common symptoms data available in the group that received complement inhibitory therapy vs. those untreated.

Author response and action taken: Thank you for your comment regarding PNH‑related symptoms. As with PNH classification, PMSI data related to symptoms are limited and are likely to capture only the most severe cases, namely those requiring hospitalization. Moreover, even in these severe cases, hospital stays may be primarily coded as PNH, without detailed information on the associated symptoms themselves. Finally, C5 inhibitor therapy is dispensed to patients by clinical experts, based on clinical symptoms and biological data (PNH clone size, hemolysis…), implying that untreated patients could remain under active surveillance without therapy, and not present with severe symptoms identifiable in PMSI. These points have been further addressed in the Discussion section.

4) Switching from eculizumab to ravulizumab is well described but interpretation is limited by the absence of clinical data. Is there any observational data?

Author response and action taken: Thank you for your comment regarding the reasons for switching between C5 inhibitors. We would like to emphasize that the PMSI database provides limited information to directly assess the motivations underlying the choice between therapies. Nevertheless, several hypotheses can be proposed. First, the study period spans from 2018 to 2022, whereas ravulizumab became available in 2020. The study therefore captures the progressive implementation of ravulizumab in clinical practice, which may be associated with a novelty or adoption effect bias. Second, ravulizumab is administered once every two months, compared with once every two weeks for eculizumab. Among patients with stable disease, switching to ravulizumab may reduce injection frequency, limit treatment burden, decrease injection fatigue, decrease related costs, and ultimately improve quality of life. In addition, the development of newer therapies with subcutaneous administration may further reduce the overall impact of treatment on patients’ daily lives. Only few manuscripts, and mostly case reports, describe the use of ravulizumab among patients who switched from eculizumab. These manuscripts mostly describe ravulizumab effectiveness and tolerance, rather than reasons motivating the switch itself. These considerations have been further developed in the Discussion section, and sources were added.

5) The sunburst figure is informative but would benefit from additional explanation or a complementary tabular summary.

Author response and action taken: Thank you for your comment regarding the readability of the sunburst figure. We have added a caption to the figure to better explain the results and to clarify how the figure should be interpreted.
